# COX-2 promotes the osteogenic potential of BMP9 through TGF-β1/p38 signaling in mesenchymal stem cells

**DOI:** 10.18632/aging.202825

**Published:** 2021-04-04

**Authors:** Yan Deng, Ling Li, Jia-Hui Zhu, Pei-Pei Li, Yi-Xuan Deng, Hong-Hong Luo, Yuan-Yuan Yang, Bai-Cheng He, Yuxi Su

**Affiliations:** 1 Department of Orthopedics, Children's Hospital of Chongqing Medical University, Chongqing 400014, China; 2 Chongqing Key Laboratory of Pediatrics, Chongqing Medical University, Chongqing 400014, China; 3 Ministry of Education Key Laboratory of Child Development and Disorders, Chongqing Medical University, Chongqing 400014, China; 4 National Clinical Research Center for Child Health and Disorders, Chongqing Medical University, Chongqing 400014, China; 5 China International Science and Technology Cooperation Base of Child Development and Critical Disorders, Chongqing Medical University, Chongqing 400014, China; 6 Children’s Hospital of Chongqing Medical University, Chongqing 400014, China; 7 Key Laboratory of Biochemistry and Molecular Pharmacology of Chongqing, Chongqing Medical University, Chongqing 400016, China; 8 Department of Pharmacology, School of Pharmacy, Chongqing Medical University, Chongqing 400016, China

**Keywords:** BMP9, TGF-β1, COX-2, osteogenic differentiation, p38 MAPK

## Abstract

This study investigated the effects of transforming growth factor-β1 (TGF-β1) and cyclooxygenase-2 (COX-2) on bone morphogenetic protein 9 (BMP9) in mesenchymal stem cells (MSCs). We found that BMP9 increased mRNA levels of TGF-β1 and COX-2 in C3H10T1/2 cells. BMP9-induced osteogenic markers were enhanced by TGF-β1 and reduced by TGF-βRI-specific inhibitor LY364947. BMP9 increased level of p-Smad2/3, which were either enhanced or reduced by COX-2 and its inhibitor NS398. BMP9-induced osteogenic markers were decreased by NS398 and it was partially reversed by TGF-β1. COX-2 increased BMP9-induced osteogenic marker levels, which almost abolished by LY364947. BMP9-induced bone formation was enhanced by TGF-β1 but reduced by silencing TGF-β1 or COX-2. BMP9’s osteogenic ability was inhibited by silencing COX-2 but partially reversed by TGF-β1. TGF-β1 and COX-2 enhanced activation of p38 signaling, which was induced by BMP9 and reduced by LY364947. The ability of TGF-β1 to increase the BMP9-induced osteogenic markers was reduced by p38-specific inhibitor, while BMP9-induced TGF-β1 expression was reduced by NS398, but enhanced by COX-2. Furthermore, CREB interacted with Smad1/5/8 to regulate TGF-β1 expression in MSCs. These findings suggest that COX-2 overexpression leads to increase BMP9’s osteogenic ability, resulting from TGF-β1 upregulation which then activates p38 signaling in MSCs.

## INTRODUCTION

BMP9 is one of major osteogenic induction factors with promising clinical applications [1]; however, some limitations of the osteogenic potential of BMP9, such as uncontrolled adipogenesis differentiation and longer duration for bone matrix maturation [2]. Therefore, to improve the clinical application of BMP9, its osteogenic capacity needs to be enhanced. To date, the exact biological mechanisms involved in BMP9-induced osteogenesis is still under investigation, even though various signaling factors, such as Notch, insulin-like growth factors, Hedgehog, Wnts, epidermal growth factor, all-trans retinoic acid, heme oxygenase 1, and the long non-coding RNA H19 [3–9], have been implicated in this process.

It’s well known that transforming growth factor β1(TGF-β1) is one of critical factors in regulating multiple physiological functions [10]; however, the mechanisms by which TGF-β1 affects bone metabolism remain controversial. For example, TGF-β1 can increase the bone forming ability of mesenchymal stem cells (MSCs) [11], by committing the progenitors either to adipogenic or to osteoblastic lineages via the reorganization of the cytoskeleton [12]. However, during the BMP9-induced osteoblastic commitment in MSCs, it was shown that TGF-β1 has biphasic effects [13]. The differential effects of TGF-β1 on bone metabolism may be caused by several factors, including the cell types concentrations of TGF-β1, and the cellular microenvironment. It remains unclear how TGF-β1 regulates the osteogenic activity of BMP9; moreover, the relationship between BMP9 and TGF-β1 in progenitor cells during the process of osteogenic commitment requires further study.

Cyclooxygenases (COXs), comprising COX-1, COX-2, and COX-3, are the enzymes that catalyze the production of prostaglandins (PGs) and are expressed in cells. It has been shown that COX-2 is induced in response to inflammation; it is also upregulated by TGF-β1 [14]. COX-2 is also associated with the regulation of bone metabolism: the osteogenic ability of bone MSCs is attenuated or delayed if COX-2 is knocked out [15]. We previously showed that COX-2 is induced by BMP9 in MSCs and, in turn, increases the osteogenic ability of BMP9 via the canonical or non-canonical BMP/Smad, PI3K/Akt, and Wnt/β-catenin signaling pathways [16–18]. However, the exact mechanism underlying the cross-talk between COX-2 and TGF-β1 during the osteogenesis induced by BMP9 remains unknown.

Here, we elucidated the role of TGF-β1 in the effect of COX-2 on strengthening the BMP9-committed osteogenic differentiation in MSCs.

## RESULTS

### The effect of BMP9 on the expression of COX-2 and TGF-β1 in MSCs

**Figure 1:**
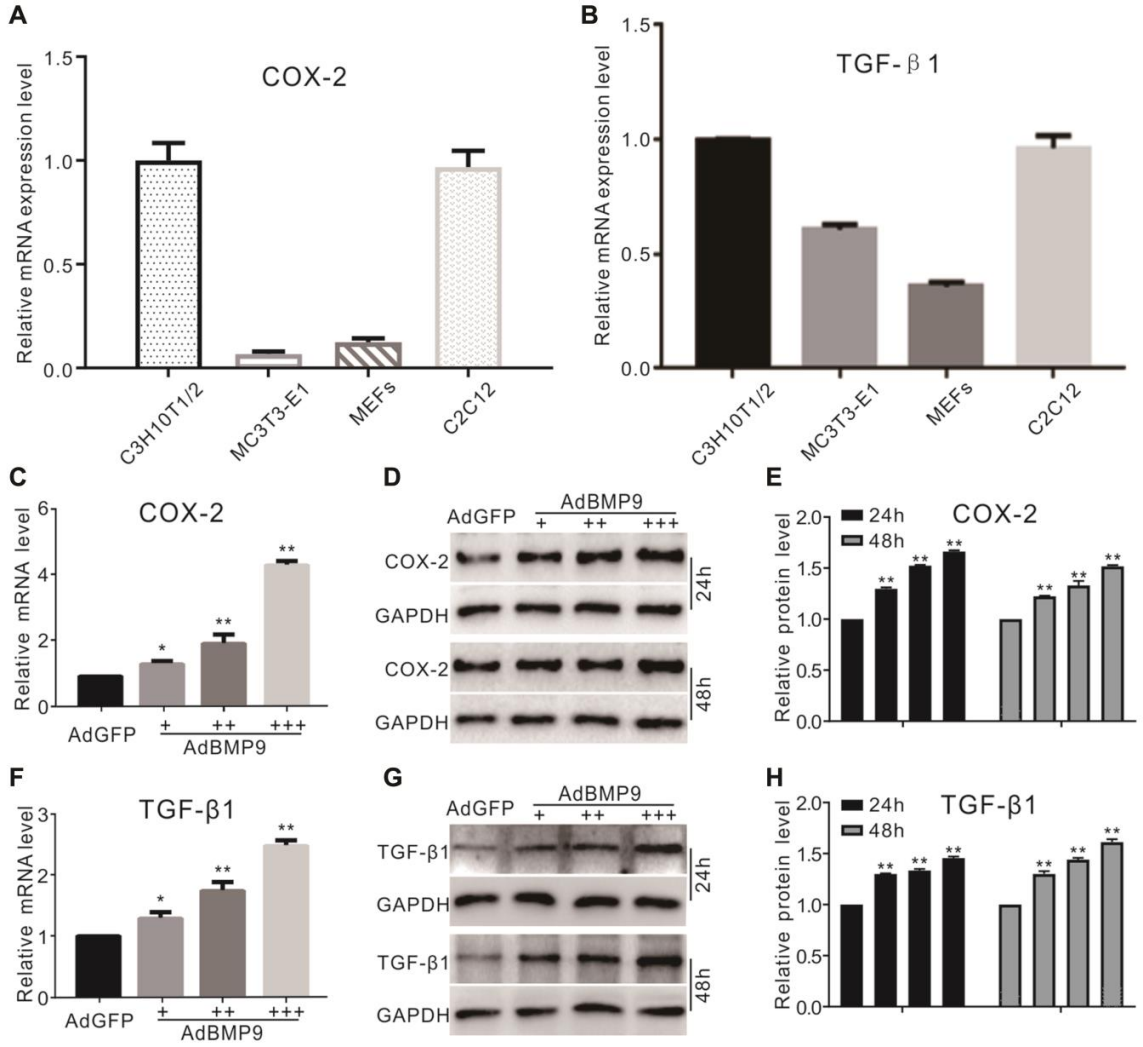
**The effect of BMP9 on TGF-β1 and/or COX-2 in MSCs.** (**A**) and (**B**) show real-time PCR analysis results of COX-2 or TGF-β1 expression in several kinds of progenitor cells. (**C**) COX-2 mRNA expression induced by BMP9 (real-time PCR). (**D**) Western blotting of the BMP9-induced protein level of COX-2. (**E**) Quantification of western blots showing the effect of BMP9 on COX-2 protein levels. (**F**) Real-time PCR data show that TGF-β1 was induced by BMP9. (**G**) Western blotting results show that TGF-β1 was induced by BMP9. (**H**) Quantification of western blotting shows that TGF-β1 was induced by BMP9. All experiments were performed in C3H10T1/2 cells. ^“+”^, ^“++”^ and ^“+++”^ indicate increasing titer of recombinant adenovirus; ^“*”^*p* < 0.05 and ^“**”^*p* < 0.01.

The connection between BMP9 and TGF-β1 during osteogenesis remains unclear. COX-2, a downstream target of BMP9, can promote BMP9-induced osteogenesis; however, the detailed mechanism of COX-2 function needs to be further studied. Real-time PCR analysis results show the mRNA of COX-2 or TGF-β1 are detectable in several progenitor cells (Figure 1A and 1B). In C3H10T1/2 cells, our real-time PCR and western blotting results indicated that BMP9 could induce COX-2 expression obviously (Figure 1C–1E). At the same time, BMP9 also increased the mRNA and protein levels of TGF-β1 (Figure 1D and 1F). These data suggested that COX-2, together with TGF-β1, might be associated with the process of osteogenic lineage commitment in MSCs via BMP9 stimulation.

### The effect of TGF-β1 on BMP9-induced osteogenic marker levels

**Figure 2:**
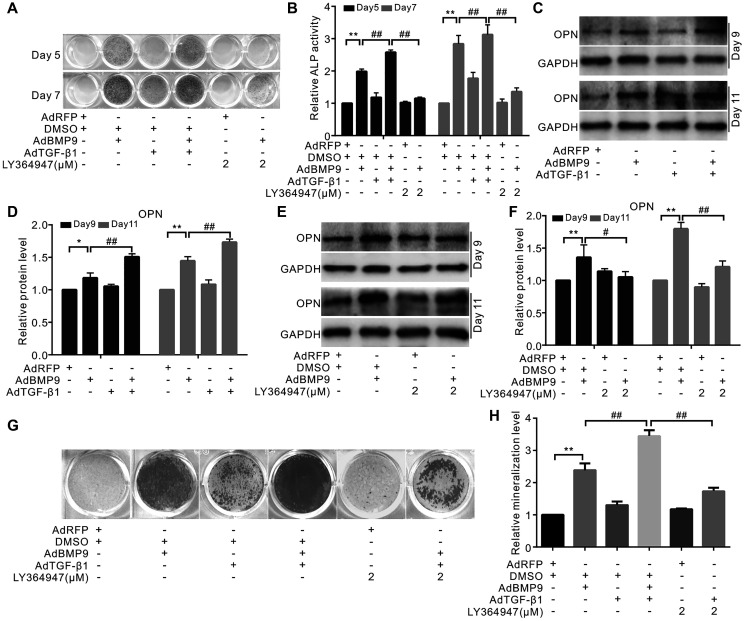
**Effects of TGF-β1 on BMP9-induced osteogenic makers in C3H10T1/2 cells.** (**A**) ALP staining shows the effect of TGF-β1 or LY364947 (TGF-βRI inhibitor) on the ALP activity induced by BMP9. (**B**) Quantification results of ALP assay show the BMP9-induced ALP activities was affected by TGF-β1 and/or LY364947. (**C**) Western blotting results show OPN was affected by TGF-β1. (**D**) Quantification results of western blot assay show OPN was affected by TGF-β1 and/or BMP9. (**E**) Western blotting analysis showed OPN level was affected by BMP9 and/or LY364947. (**F**) Quantification results of western blot assay show OPN was affected by BMP9 and/or LY364947. (**G**) Alizarin Red S assay shows mineralization was affected by TGF-β1, LY364947, and/or BMP9. (**H**) Quantification results of Alizarin Red S assay show that mineralization was affected by TGF-β1, LY364947, and/or BMP9. ^“**”^*p* < 0.01, ^“#”^*p* < 0.05, and ^“##”^*p* < 0.01.

TGF-β1 slightly increased the ALP activity and significantly enhanced the effect of BMP9 on ALP activity. Interestingly, a specific inhibitor of TGF-βRI LY364947 showed no effect on ALP activity, but substantially inhibited the ALP activity induced by BMP9. (Figure 2A and 2B). There was no considerable change upon increasing the level of osteopontin (OPN) in the presence of TGF-β1; however, TGF-β1 could potentiate BMP9-induced OPN (Figure 2C and 2D). The inhibition of TGF-βRI decreased the level of OPN induced by BMP9 (Figure 2E and 2F). Similar results were observed in the mineralization assay: TGF-β1 markedly increased the mineralization induced by BMP9, and inhibition of TGF-βRI reduced this mineralization (Figure 2G and 2H). These results showed that TGF-β1 could enhance the osteogenic potential of BMP9; however, the detailed mechanism of COX-2 and TGF-β1 association is not clear.

### The effect of TGF-β1 and/or COX-2 on the osteogenic markers’ level induced by BMP9

**Figure 3:**
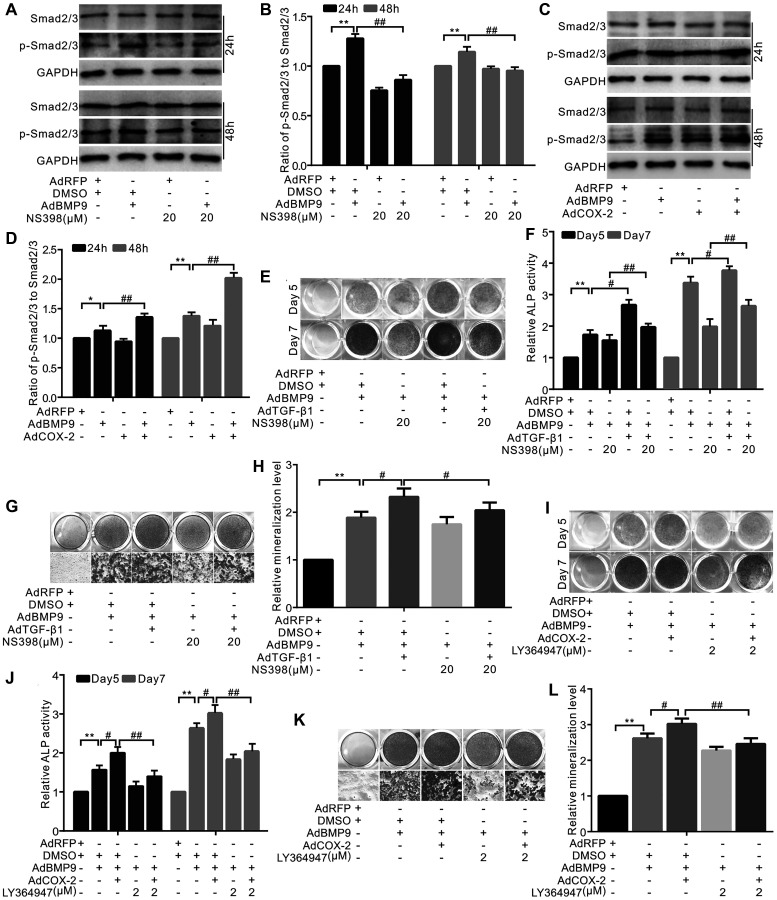
**The effect of TGF-β1 and/or COX-2 on osteoblastic markers induced by BMP9 in C3H10T1/2 cells.** (**A**) Western blotting shows that NS-398 and/or BMP9 affect Smad2/3 and p-Smad2/3 (phosphorylated Smad2/3). (**B**) Quantification results of Western blots assay shows Smad2/3 or p-Smad2/3 was affected by NS-398 and/or BMP9. (**C**) Western blotting shows the level of Smad2/3 or p-Smad2/3 was affected by COX-2 and/or BMP9. (**D**) Quantification results of western blot assay shows the level of Smad2/3 and/or p-Smad2/3 was affected by COX-2 and/or BMP9. (**E**) ALP staining shows the effect of NS-398, TGF-β1, and/or BMP9 on ALP activity. (**F**) Quantification of ALP staining shows that ALP activities were affected by NS-398, TGF-β1, and/or BMP9. (**G**) Alizarin Red S staining shows that the mineralization was affected by NS-398, TGF-β1, and/or BMP9. (**H**) Quantification results of Alizarin Red S assay shows that mineralization was affected by NS-398, BMP9, and/or TGF-β1. (**I**) ALP assay shows the BMP9-induced ALP activities was affected by COX-2 and LY364947. (**J**) Quantification of ALP assay shows that ALP activities were affected by COX-2, LY364947, and/or BMP9. (**K**) Alizarin Red S staining shows that the mineralization was affected by COX-2, LY364947, and/or BMP9. (**L**) Quantification results of Alizarin Red S staining shows that mineralization was affected by COX-2, LY364947, and/or BMP9. LY364947: TGF-βRI inhibitor; NS-398: COX-2 inhibitor. ^“**”^*p* < 0.01, ^“#”^*p* < 0.05, and ^“##”^*p* < 0.01.

Our results demonstrated that BMP9 or NS-398 (COX-2 inhibitor) did not affect the total level of Smad2/3; however, BMP9 increased the level of p-Smad2/3, which, in turn, was almost abolished by NS-398 (Figure 3A and 3B). However, COX-2 enhanced the BMP9’s effect on increasing p-Smad2/3 level (Figure 3C and 3D). TGF-β1 increased the effect of BMP9 on ALP activity, which was partially abolished by NS-398 (Figure 3E and 3F). The BMP9-induced mineralization was enhanced by TGF-β1 and reduced by NS-398; the inhibitory effect of NS-398 on BMP9 was almost reversed by TGF-β1 (Figure 3G and 3H). The BMP9-induced ALP activity was enhanced by COX-2, which, in turn, was inhibited by the TGF-βRI-specific inhibitor (Figure 3I and 3J). Similar results were also obtained in BMP9-induced bone mineralization experiments (Figure 3K and 3L). These data suggest that TGF-β1 along with COX-2 promotes the BMP9-induced osteogenic differentiation.

### The effect of TGF-β1 and/or COX-2 on the osteogenesis induced by BMP9

**Figure 4:**
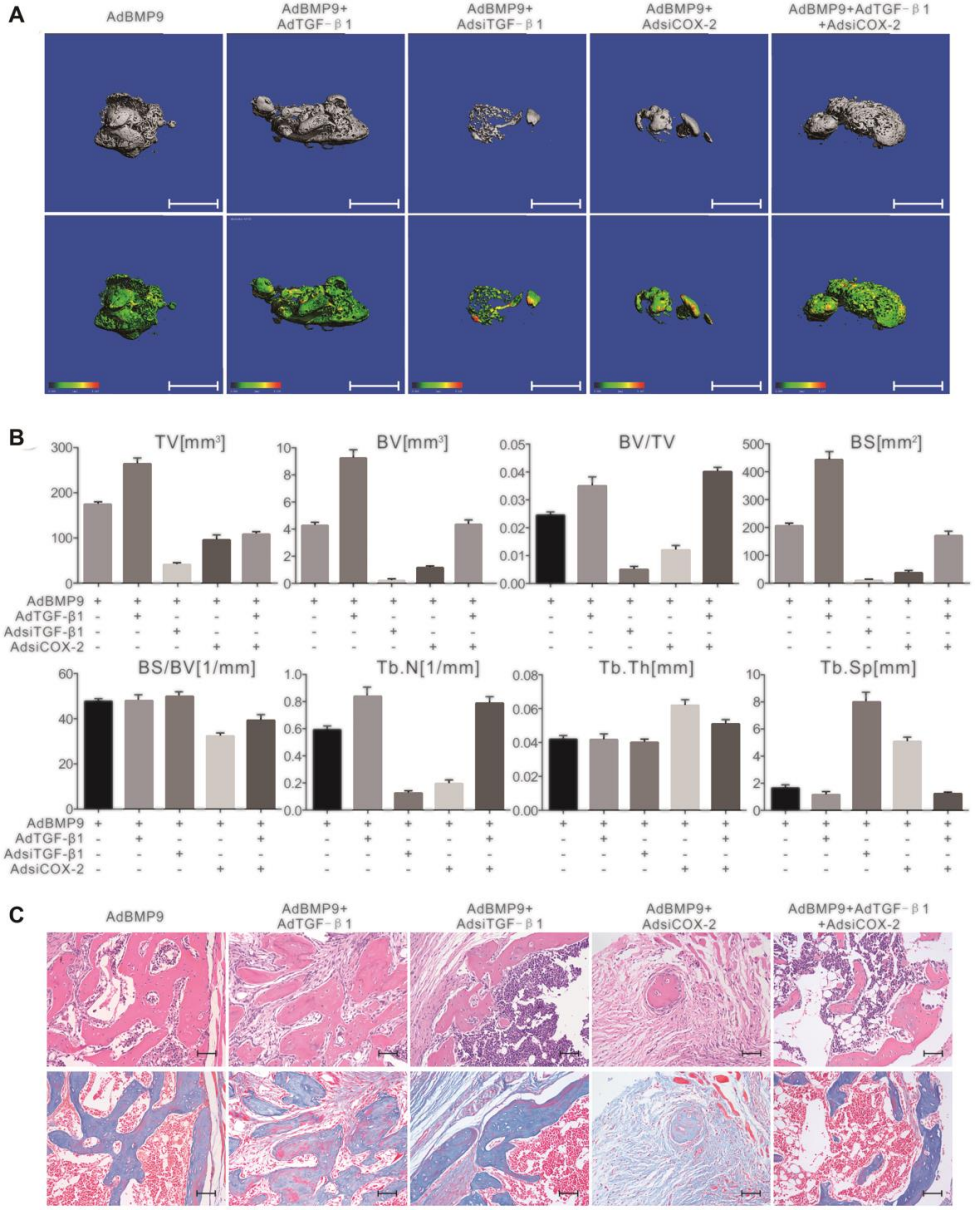
**The effect of TGF-β1 and COX-2 knockdown on the osteogenesis induced by BMP9 in C3H10T1/2 cells.** (**A**) The 3D-reconstruction of μ-CT shows that bone formation was affected by TGF-β1, COX-2 knockdown, and/or BMP9. Scale bar = 2.5 mm. (upper panel: reconstructed bone samples; lower panel: heat map showing the bone density of the samples). (**B**) Quantification of μ-CT shows that the BMP9-induced bone formation was affected by TGF-β1 and COX-2. (**C**) Histological staining shows the osteogenesis ability was affected by TGF-β1 and/or COX-2. Scale bar = 100 μm. (Upper: H&E staining; lower: Masson trichrome staining).

The 3D reconstruction of μ-CT scan demonstrated that TGF-β1 increased the bone volume induced by BMP9, while TGF-β knockdown had the opposite effect. The bone volume induced by BMP9 was also decreased in response to COX-2 downregulation, but was almost reversed by TGF-β1 (Figure 4A). Quantification of μ-CT analysis showed similar results (Figure 4B). TGF-β1 promoted the growth of trabecular bone induced by BMP9; however, the knockdown of TGF-β1 or COX-2 decreased this effect. Interestingly, TGF-β1 partly reversed the effect of silencing COX-2 on the osteogenesis induced by BMP9 (Figure 4C). This experiment confirmed that TGF-β1, along with COX-2 enhances the osteogenic ability of BMP9 in MSCs.

### The role of p38 signaling in the osteoblastic differentiation induced by BMP9 and/or TGF-β1 in C3H10T1/2 cells

**Figure 5:**
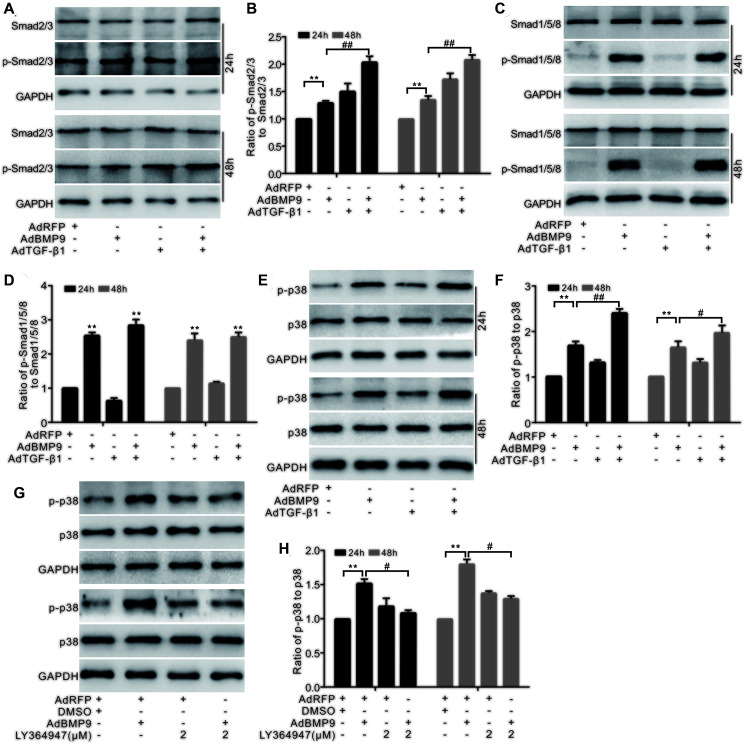
**The effect of BMP9 and/or TGF-β1 on the p38 signaling in MSCs.** (**A**) Western blotting shows that the level of Smad2/3 or p-Smad2/3 was affected by TGF-β1 and/or BMP9. (**B**) Quantification results of western blots shows that the level of Smad2/3 or p-Smad2/3 was affected by COX-2 and/or BMP9. (**C**) Western blotting shows the effects of TGF-β1 and BMP9 on the levels of Smad1/5/8 and p-Smad1/5/8. (**D**) Quantification of western blots shows that the levels of Smad1/5/8 and p-Smad1/5/8 were affected by BMP9. (**E**) Western blotting shows the effect of TGF-β1 and/or BMP9 on the level of p38 and p-p38. (**F**) Quantification results of western blots shows the level p38 and p-p38 was affected by TGF-β1 and/or BMP9. (**G**) Western blotting shows that the level of p38 and p-p38 was affected by LY364947 and/or BMP9. (**H**) Quantification of western blots shows that the level of p38 and p-p38 was affected by LY364947 and/or BMP9. LY364947: TGF-βRI specific inhibitor. ^“**”^*p* < 0.01, ^“#”^*p* < 0.05, and ^“##”^*p* < 0.01.

Western blotting showed that BMP9 had no effect on Smad2/3 and p-Smad2/3; however, TGF-β1 increased the level of p-Smad2/3. Furthermore, p-Smad2/3 levels were synergistically elevated by BMP9 and TGF-β1 (Figure 5A and 5B). BMP9 increased the level of p-Smad1/5/8; however, there was no considerable change in p-Smad1/5/8 levels in response to TGF-β1, even in combination with BMP9 (Figure 5C and 5D). Based on these results, we hypothesized that TGF-β1 was not involved in the BMP/Smad signaling during the BMP9-induced osteogenetic differentiation. Western blotting showed that both BMP9 and TGF-β1 increased p38 phosphorylation, and that this effect of BMP9 on p-p38 was further enhanced by TGF-β1, but decreased by a TGF-βRI-specific inhibitor (Figure 5E–5H). The above experiments further confirmed that TGF-β1 may act through p38 signaling to enhance the osteogenic differentiation of BMP9.

### Role of p38 in handing the effect of TGF-β1 and/or COX-2 to promote BMP9’s osteoblastic potential in MSCs

**Figure 6:**
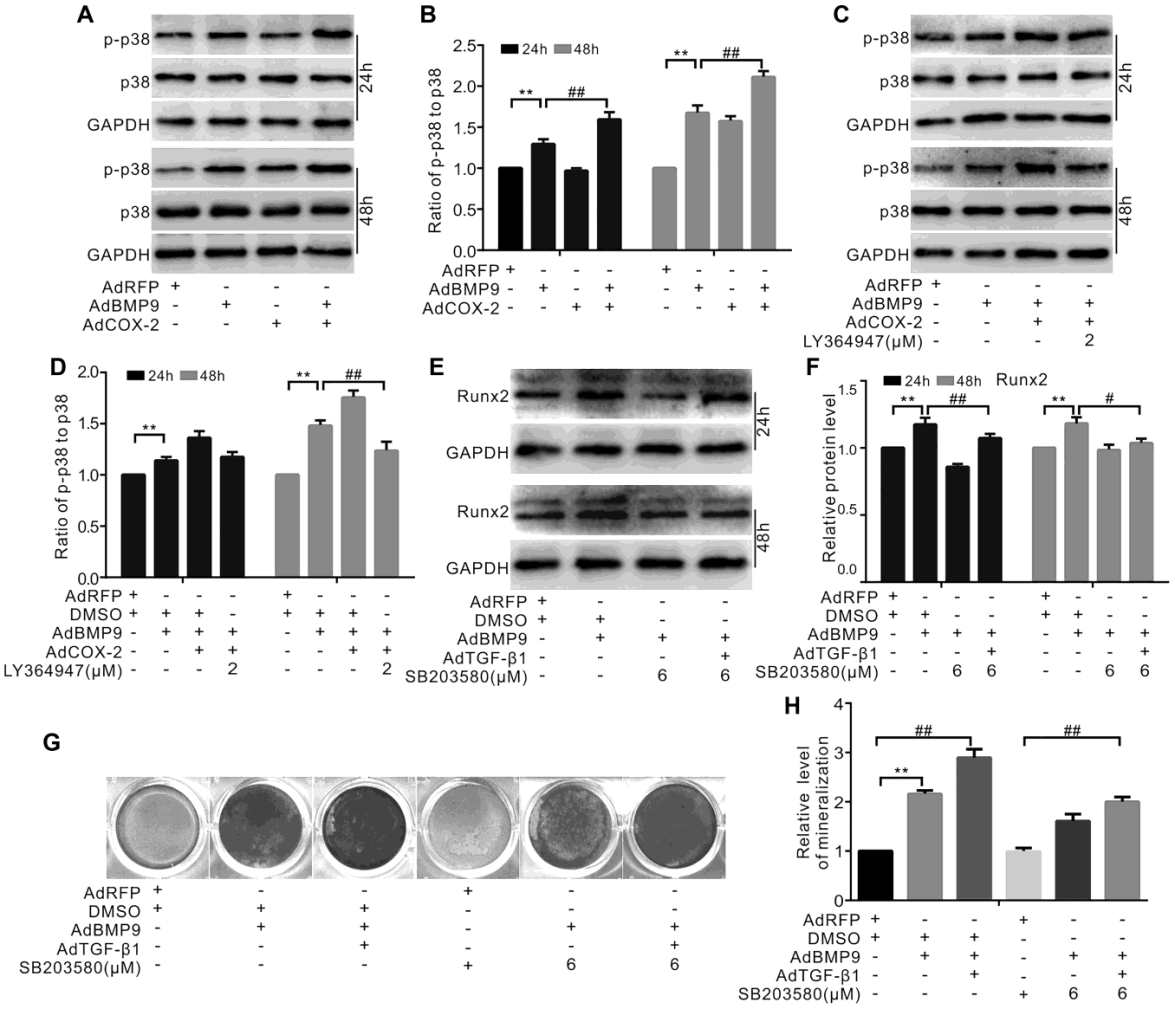
**The role of p38 in mediating the effect of COX-2 and/or TGF-β1 on enhancing the osteogenic potential of BMP9 in MSCs**. (**A**) Western blotting shows that the levels of p38 and p-p38 were affected by BMP9 and/or COX-2. (**B**) Quantification of western blots shows that the level of p38 and p-p38 was affected by BMP9 and/or COX-2. (**C**) Western blotting shows that the level of p38 and p-p38 was affected by LY364947, COX-2, and/or BMP9. (**D**) Quantification results of western blots assay shows that the level of p38 and p-p38 was affected by LY364947, COX-2, and BMP9. (**E**) Western blotting shows the potential effect of BMP9, TGF-β1, and/or SB203580 on Runx2. (**F**) Quantification results of western blots shows the level of Runx2 was affected by BMP9, TGF-β1, and/or SB203580. (**G**) Alizarin Red S staining shows that the mineralization was affected by BMP9, TGF-β1, and/or SB203580 (day 20). (**H**) Quantification results of Alizarin Red S assay shows that mineralization was affected by BMP9, TGF-β1, and/or SB203580. SB203580: p38 MAPK specific inhibitor; LY364947: TGF-βRI specific inhibitor. ^“**”^*p* < 0.01, ^“#”^*p* < 0.05, and ^“##”^*p* < 0.01.

The data showed that p38 phosphorylation was stimulated separately by BMP9 or COX-2 and that the combination of BMP9 and COX-2 had a synergistic effect. However, neither BMP9 nor COX-2 exhibited any effect on the total level of p38 (Figure 6A and 6B). Furthermore, the synergistic effect of COX-2 on BMP9-induced p-p38 levels could be abolished by the TGF-βRI-specific inhibitor LY364947 (Figure 6C and 6D). Furthermore, the specific inhibitor of p38 SB203508 reduced the level of Runx2 induced by BMP9, but TGF-β1 partly reversed this effect (Figure 6E and 6F). At the same time, BMP9-induced bone mineralization was enhanced by TGF-β1, but was reduced by a p38-specific inhibitor. TGF-β1 partially reversed the effect of the p38-specific inhibitor on BMP9-induced mineralization (Figure 6G and 6H). According to these results, we hypothesized that TGF-β1 and/or COX-2 may increase the osteogenic function of BMP9 by activating p38-MAPK signaling.

### Effects of COX-2 and BMP9 on TGF-β1

**Figure 7:**
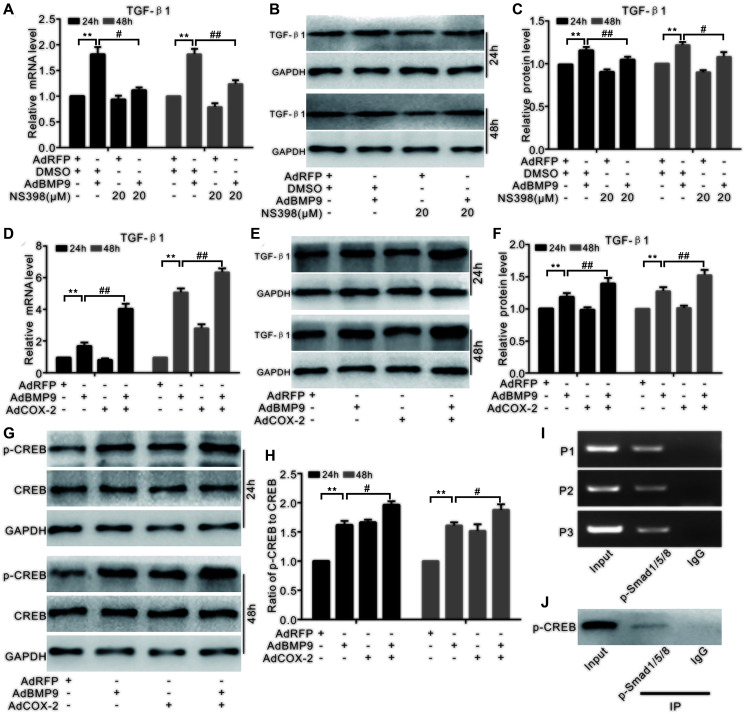
**The effect of COX-2 and BMP9 on TGF-β1 expression in C3H10T1/2 cells.** (**A**) Real-time PCR analysis shows the mRNA level of TGF-β1 was affected by BMP9 and/or NS-398. (**B**) Western blotting shows that the level of TGF-β1 was affected by BMP9 and/or NS-398. (**C**) Quantification of western blots shows that TGF-β1 level was affected by BMP9 and/or NS-398. (**D**) Real-time PCR assay shows that TGF-β1 mRNA expression was affected by BMP9 and/or COX-2. (**E**) Western blotting shows that TGF-β1 level was affected by BMP9 and/or COX-2. (**F**) Quantification of the western blots shows that TGF-β1 level was affected by BMP9 and/or COX-2. (**G**) Western blotting shows that the level of CREB and p-CREB was affected by BMP9 and/or COX-2. (**H**) Quantification of the western blots shows that the levels of CREB and p-CREB was affected by BMP9 and/or COX-2. (**I**) ChIP assay shows the enrichment of p-Smad1/5/8 at the TGF-β1’s putative promoter region. (**J**) IP assay shows p-CREB may interact with p-Smad1/5/8. NS-398: COX-2 specific inhibitor; ^“**”^*p* < 0.01, ^“#”^*p* < 0.05, and ^“##”^*p* < 0.01.

Real-time PCR analysis showed that BMP9 can stimulate TGF-β1 mRNA expression. NS-398 treatment showed no significant effect on the *TGF-β1* mRNA expression; however, it reduced the effect of BMP9 on *TGF-β1* mRNA expression (Figure 7A). Furthermore, BMP9 increased TGF-β1 protein levels, but this effect was significantly reversed by NS-398 (Figure 7B and 7C). COX-2 increased the *TGF-β1* mRNA levels, while BMP9 also had a similar effect on the *TGF-β1* mRNA expression in C3H10T1/2 cells (Figure 7D). In addition, BMP9 increased the protein level of TGF-β1; however, COX-2 had no effects on TGF-β1, even though it could significantly enhance the effect of BMP9 on TGF-β1 (Figure 7E, 7F). BMP9 increased p-CREB level, which was also enhanced by COX-2 (Figure 7G, 7H). The ChIP assay showed that p-Smad1/5/8 was presented in TGF-β1’s promoter region (Figure 7I), and the IP assay indicated that p-CREB interacted with p-Smad1/5/8 also (Figure 7J). These experiments suggested that the effect of BMP9 on up-regulating TGF-β1 may be strengthened by COX-2 through the association of p-CREB and p-Smad1/5/8.

## DISCUSSION

TGF-β1 is a secreted protein involved in skeletal maintenance and development; however, its effects on osteogenic differentiation in multipotential progenitor cells remain controversial. Therefore, the molecular mechanisms involved in this physiological process need to be further investigated. In this study, we found that TGF-β1 promoted the osteogenic capacity of BMP9 by enhancing the activation of p38 signaling. At the same time, COX-2 increased BMP9’s osteogenic potential by inducing TGF-β1 expression, possibly related with the association of CREB and Smad1/5/8 in MSCs.

The TGF-β family includes three isoforms, which are secreted by various cell types and play a critical role in the immune response, cell differentiation, and proliferation [19]. TGF-β acts by binding to one of its corresponding receptors (TGF-βRI and TGF-βRII) and affects the expression of downstream genes in a Smad2/3-dependent manner. TGF-β1 was first identified in human platelets and has a potential role in wound healing [20]. It has been established that TGF-β1 plays an important role in regulating immune system; however, this role depends on the cell type and the developmental stage [21]. BMP, as osteogenic factor, was discovered by Marshall R. Urist in 1965 [22]. So far, at least 15 BMPs have been identified in humans. BMP2 or BMP7 has already been approved for clinical use in orthopedic applications, such as non-unions, spinal fusions, and oral surgery [2, 23]. Although several BMPs, for example BMP-2 and BMP-4, possess excellent osteogenic properties, their levels remain very low in the peripheral blood after a bone fracture. Unlike BMPs, TGF-β1 is markedly increased in peripheral blood within two weeks after a fracture [24], recruiting bone MSCs to the fracture site to promote bone formation [25]. Meanwhile, TGF-β1 is involved in regulating bone remodeling and growth during development, as well as the maintenance of MSCs [19, 26–29]. TGF-β1 also increases osteogenic and chondrogenic differentiation in human dental pulp-derived stromal cells and murine bone marrow stromal cells [30, 31]. However, TGF-β1 exerts biphasic effects during the BMP9-induced osteogenic differentiation: TGF-β1 promotes osteogenic potential at lower concentrations, but is inhibitory at high concentrations [13]. Our pilot experiments also showed that TGF-β1 could inhibit BMP9-induced osteogenic differentiation at high concentration (data not shown). In addition to the TGF-β1 concentration, its effects also depend on the cell type and microenvironment.

BMP9, identified originally in the mouse liver [32], is involved in the regulation of glucose balance and iron metabolism [33, 34]. In addition, BMP9 has a strong osteogenic potential, with a higher osteogenic differentiation ability compared to BMP2 or BMP7 [2]; therefore, BMP9 could be used as a promising alternative in bone tissue engineering. Although this ability can be regulated by various signaling pathways and non-coding RNA [5–7, 9], the mechanism of possible interaction between BMP9 and TGF-β1 remains unclear. Here, TGF-β1 was detectable in other several progenitor cells and upregulated by BMP9 in MSCs. This evidence suggested that TGF-β1 was associated with the osteogenic function of BMP9. Next, we demonstrated that TGF-β1 promoted the osteogenic function of BMP9 in MSCs both *in vitro* and *in vivo*. However, mechanisms of how TGF-β1 facilitates BMP9 osteogenic function and how BMP9 regulates the expression of TGF-β1 in MSCs remains unclear.

COX-2, also termed as prostaglandin synthase-2, is a major pro-inflammatory factor. Selective inhibitors, such as celecoxib, are usually prescribed as analgesics because they have no obvious gastrointestinal side effects [35, 36]. In addition to its role as a proinflammatory cytokine, COX-2 is also involved in the regulation of bone homeostasis. Knockouts of COX-2 display delayed bone fracture healing and decreased osteogenic potential of MSCs. Interestingly, there was no such effect in COX-1 knockout mice [15]. COX-2 is also constitutively expressed in the trabeculae, periosteum, or endosteum, and its ability to induce osteogenic differentiation may be mediated through its interaction with PTEN [17, 37].

COX-2-specific inhibitors are administered as pain killers after surgery. Furthermore, heterotopic ossification after hip arthroscopic surgery could be reduced by a COX-2 selective inhibitor, such as etodolac [38]. Administration of selective COX-2 inhibitor may result in a higher risk of nonunion in patients with bone fractures [39]. This evidence supports the finding that COX-2 is associated with the regulation of bone metabolism. We have already shown that BMP9 induces COX-2 expression in MSCs, while the silence of COX-2 greatly attenuates the osteogenic-inducing ability of BMP9. Furthermore, COX-2 may increase this function of BMP9 by enhancing BMP/Smad and Wnt/β-catenin signaling [16, 40]. However, mechanisms of how COX-2 modulates the osteogenic function of BMP9 remains unclear.

To date, the precise mechanism of COX-2 and TGF-β1’s action in the BMP9-induced osteoblastic differentiation in MSCs remains unknown. Several studies have shown that TGF-β1 can induce or suppress COX-2 in mesangial or lung cancer cells [41, 42]. Since BMP9 increases TGF-β1 and COX-2 levels simultaneously in MSCs, we speculated that TGF-β1 may interact with COX-2 to regulate the osteogenic function BMP9. In our experiments, we found that TGF-β1 elevated the levels of osteogenic markers induced by BMP9, which were, in turn, reduced by NS-398, a COX-2 inhibitor. However, the effects of NS-398 on BMP9-induced osteogenic markers were almost abolished by TGF-β1. Levels of BMP9-induced osteogenic markers were enhanced by COX-2, but were significantly reduced via the inhibition of TGF-βRI. These results suggested that TGF-β1 and/or COX-2 may partially facilitate BMP9’s osteogenic function in MSCs.

BMP9 exerts its physiological function not only via BMP/Smad signaling (Smad1/5/8, complex with Smad4), but also via non-canonical signaling, such as PI3K or MAPK. MAPKs, include Erk, JNK, and p38, the protein kinases that regulate cell survival, apoptosis, proliferation, and differentiation, respectively [43]. In periodontal ligament stem cells, Erk1/2 and p38 regulates osteogenic differentiation induced by BMP9 [44]. TGF-β1 binding to its receptor activates a Smad2/3-dependent pathway; however, it can also act via non-Smad2/3-dependent signaling, for example, via p38. In human synovial fibroblasts, FOXO3 is induced by TGF-β1 through the p38 pathway [45]. The TGF-βR/p38 pathway is also involved in the CD44 regulation of α-SMA expression in murine skin fibroblasts [46]. In pancreatic carcinoma cells, TGF-β1 also induces cell migration in a p38-dependent manner [47]. Furthermore, in pancreatic cancer, COX-2 can promote angiogenesis in the EGFR/p38-dependent manner [48], while in chronic obstructive pulmonary disease, MSC-induced airway inflammation and emphysema are partially mediated by silencing *COX-2* via the p38 and ERK pathways [49]. Therefore, TGF-β1 may also regulate the osteogenic potential of BMP9 via p38 signaling. Interestingly, we found that TGF-β1 enhanced the effect of BMP9 on the activation of Smad2/3, but not Smad1/5/8. The effect of BMP9 on the activation of p38 signaling was enhanced by TGF-β1, while TGF-βRI-specific inhibitors reduced the effect of BMP9 on the activation of p38 in MSCs. The specific inhibitor of p38 inhibited the BMP9-induced expression of osteogenic differentiation markers, which was elevated by TGF-β1. Further analysis indicated that COX-2 increased the activation of p38 signaling in a TGF-β1-dependent manner. Thus, the effect of TGF-β1 on the osteogenic function of BMP9 could be regulated through p38 signaling, and TGF-β1 could also regulate the BMP9-induced osteogenic differentiation along with COX-2. CREB is one of the important signaling pathways for COX-2 to perform its physiological function. We then found that CREB correlated with Smad1/5/8 to regulate TGF-β1 in MSCs.

Taken together, this study indicated that TGF-β1 can partly regulate the effect of COX-2 on enhancing the BMP9-induced osteoblastic differentiation through p38 signaling. COX-2 may potentiate the ability of BMP9 to upregulate TGF-β1 by facilitating the interaction of CREB and Smad1/5/8.

## MATERIALS AND METHODS

### Cell culture and chemicals

C3H10T1/2 cells used in this study were purchased from the American Type Culture Collection (ATCC, Manassas VA, USA). Cells were cultured in Dulbecco’s modified Eagle’s medium (DMEM), which usually supplemented with 10% fetal bovine serum, 100 unit penicillin, and 100 μg streptomycin, at 37°C and 5% CO_2_. Antibodies against p-CREB (ab32096) and p-Smad2/3 (8828s) were purchased from Abcam (China) and Cell Signaling Technology (Shanghai, China), respectively. CREB (AF6188) and p-Smad1/5/8 (AF8313) primary antibodies were purchased from Xiangtai Biological Technology (Affinity Biosciences, China). All other primary antibodies were all purchased from the branch of Santa Cruz Biotechnology (Shanghai, China). NS-398 (N194-25MG, COX-2 inhibitor) was ordered from sigma Aldrich (China branch), LY364947 (T2048, TGF-βRI inhibitor) was bought from Targetmol (Shanghai, China), and SB203580 (S1076, p38 MAPK inhibitor) was purchased from Selleck (Shanghai, China).

### Recombinant adenoviral vector construction

The Ad-Easy system was used as a recombinant adenovirus construction system [50, 51]. Briefly, coding sequences were amplified via PCR and the PCR products for COX-2 and TGF-β1, as well as the small interfering RNA (siRNA) oligo fragments, were cloned into the adenoviral shuttle vector. Next, they were transduced into bacterial BJ5183 cells and then into HEK293 cells for the generation of recombinant adenoviruses, named AdBMP9, AdTGF-β1, AdCOX-2, AdsiTGF-β1, or AdsiCOX-2 [16]. All viruses were tagged with GFP or RFP for tracking. The control vector contained recombinant adenovirus expressing RFP only (AdRFP). Plaque-forming units (PFUs) per milliliter were determined as reported previously [50, 51], with the final adenovirus titer being 10^11^ to 10^12^.

### Alkaline phosphatase (ALP) activity assay

ALP activity was tested using the ALP analysis kit (C3206, Beyotime, Jiangsu, China) according to the instructions from manufacturer, and was measured by staining with NBT/BCIP on days 5 and 7 after the treatment. Finally, the data were acquired from scanning the plates. Experiments were repeated at least three times.

### Total RNA extraction, RT-PCR, and qPCR analyses

At the specified time points, total RNA of each sample was isolated with TRIzol (Invitrogen, USA) according to the instruction from manufacturer. The PrimeScript RT reagent (RR037A, Takara, China) was used to generate cDNA. Quantitative PCR analysis was performed after the products were diluted 5 to 10 times as stock. The reference gene used for this study is glyceraldehyde phosphate dehydrogenase (GAPDH). PCR primers used in this study are listed in Table 1.

**Table 1.** The primers used for PCR.

**Gene**	**Primer**	**Sequence (5′ →3′)**
COX-2	F	AGAAGGAAATGGCTGCAGAA
	R	GCTCGGCTTCCAGTATTGAG
TGF-β1	F	CCTGCTGCTTTCTCCCTCAA
	R	CATAGATGGCGTTGTTGCGG
GAPDH	F	ACCCAGAAGACTGTGGATGG
	R	CACATTGGGGGTAGGAACAC
TGF-β1 (ChIP)	Primer1 F	TCTCTAACGCCTCTCCTCCC
	Primer1 R	TCCATAGCGATCGAAGTGGC
	Primer2 F	GATACCATCTACAGCGGGGC
	Primer2 R	CTCATGTCCCTCCAACCCAC
	Primer3 F	GCAGTGTTCAGCCCCAAATG
	Primer3 R	CAATGCTTGGAGATGCAGCC

F: forward; R: reverse

### Western blot assay

The cells were harvested and lysed in the lysis buffer (R0020-100, Solarbio, China). The lysates were then denatured by boiling. Next, proteins were separated using sodium dodecyl sulfate-polyacrylamide gel electrophoresis and then transferred to polyvinylidene difluoride membranes following the regular protocol for western blot. Finally, the blots were developed using an ECL kit (34096, Thermo Scientific, USA) and the images were acquired using the Bio-Rad Dox imaging system. The experiments were repeated at least three times.

### Mineralization assay

To induce mineralization, the cells were cultured using complete DMEM containing osteogenic factors: 10 nM dexamethasone, 10 mM β-glycerophosphate, and 50 μg/mL ascorbic acid. Calcium precipitation was detected on day 20 of culture using Alizarin Red S staining (A5533-25G, Sigma-Aldrich, Beijing, China) and quantified as previously described [17]. The experiments were repeated at least three times.

### Chromatin immunoprecipitation assay (ChIP)

Standard ChIP analysis was performed after 30 h of infection with AdGFP or AdBMP9, as previously described [16]. The protein-DNA complexes were precipitated using the antibody against phosphorylated Smad1/5/8 (p-Smad1/5/8); antibody against rabbit IgG was used as a control. Next, the enrichment of TGF-β1 promoter fragments was analyzed using PCR. Primers’ sequence for this assay are listed in Table 1.

### Immunoprecipitation (IP) assay

After 30 h of infection, cells were lysed using the RIPA lysis buffer (R0020, Solarbio, China) containing protease and phosphatase inhibitors (B14002 and B15002, Bimake, Shanghai, China); all procedures were performed on ice. Protein G magnetic beads (S1430, NEB China group, Beijing, China) were pre-washed with 30 μL lysis buffer. Cell lysates were incubated with the antibody against phosphorylated Smad1/5/8 for 1 h at 4°C. About 15 μL of pre-washed protein G magnetic beads were then added to the lysates and incubated for 1 h at 4°C. The target complexes were collected with a magnetic stand and washed with lysis buffer. Next, 30 μL lysis buffer was used to elute the proteins from beads. Finally, the proteins were analyzed by western blot assay. Antibody against p-CREB were used for detection.

### Ectopic bone formation assay

Six-week-old nude female mice (*n* = 5/group) were used for these experiments. Nude mice were ordered from the animal center of Chongqing Medical University (Chongqing, China). This study was approved by the Institutional Animal Care and Use Committee of Chongqing Medical University. First, the C3H10T1/2 cells were cultured in 100-mm dishes. Twenty-four hours after the treatment according to the experiment design, the cells were injected into the subcutaneous of athymic nude mice (5×10^6^ cells per injection). After five weeks, all animal were euthanasia and the bone samples were collected and fixed in 10% formalin.

### Micro-computed tomographic (μ-CT) analysis

A VivaCT 40 system (SCANCO Medical AG, Switzerland) was used to analyze bone samples. Then, the3D reconstruction and quantitative analysis were performed using μ-CT 516.1 software.

### Histological staining and evaluation

Bone samples were decalcified, and then embedded in paraffin. After de-paraffinization and re-hydration, the paraffin sections were subjected with hematoxylin and eosin (H&E) staining, and Masson trichrome staining.

### Statistical analysis

The quantitative data are presented as the mean ± SD. Two-tailed Student’s *t*-test was used for comparisons between two groups. One-way analysis of variance and Tukey's *post hoc* test were used to analyze several groups. The difference was considered statistically significant if the value of p is less than 0.05.
